# Misalignment Between Public Reimbursement and Private Dental Service Provision in Romania: A Cross-Sectional Analysis of 420 Dental Practices in Three Major Municipalities

**DOI:** 10.3390/healthcare14152349

**Published:** 2026-08-01

**Authors:** Mihaela-Andreea Bohîlțea, Ana Cernega, Vlad Gabriel Vasilescu, Simona Pârvu, Marina Imre, Silviu-Mirel Pițuru

**Affiliations:** 1 Discipline Organization, Professional Legislation and Management of the Dental Office, Faculty of Dental Medicine, “Carol Davila” University of Medicine and Pharmacy, 020021 Bucharest, Romania; mihaela-andreea.bohiltea@drd.umfcd.ro (M.-A.B.); silviu.pituru@umfcd.ro (S.-M.P.); 2 Discipline of Dental Prosthesis Technology, Faculty of Dentistry, “Carol Davila” University of Medicine and Pharmacy, Dionisie Lupu Street, No. 37, District 2, 020021 Bucharest, Romania; 3 National Institute of Public Health, Faculty of Medicine, “Carol Davila” University of Medicine and Pharmacy, 050474 Bucharest, Romania; simona.parvu@umfcd.ro; 4 Discipline of Prosthodontics, Faculty of Dentistry, “Carol Davila” University of Medicine and Pharmacy, 37 Dionisie Lupu Street, District 2, 020021 Bucharest, Romania; marina.imre@umfcd.ro

**Keywords:** oral health, dental services, public reimbursement, private dental market, healthcare access, benefit basket, health policy

## Abstract

**Background/Objectives:** Romania’s National Health Insurance House (CNAS) reimbursement basket is the principal public financial-protection mechanism, yet whether it reflects the therapeutic services patients actually seek has not been tested against private-market offerings. This study compared the public basket with the private dental service portfolio, using the latter as an indirect, supply-side signal of expressed need rather than a direct measure of demand. **Methods:** In this cross-sectional study (November 2024–February 2025), the publicly listed portfolios of 420 private dental practices in Bucharest (n = 256), Cluj-Napoca (n = 104), and Iași (n = 60) were analyzed, stratified by size (0–3 vs. >3 employees). Practices were identified from CAEN 8623 records on listafirme.ro; portfolios were extracted from official websites displaying prices. Services were classified as functional or aesthetic by two independent raters (Cohen’s κ = 0.91), with disagreements resolved by consensus. **Results:** Across all municipalities and strata, approximately 53–68% of private services fell outside the CNAS basket. Critically, about 95–96% of private services were functional rather than aesthetic (4–5%), indicating that the gap reflects functional treatments patients seek and providers offer yet that remain unfunded—not elective demand. No statistically significant geographic differences emerged. Geographic location was not associated with portfolio breadth, whereas in multivariable models larger practices (>3 employees) independently offered approximately 33% more services. **Conclusions:** Because the analysis reflects advertised portfolio breadth rather than utilization, and a documented Romanian orientation toward reactive over preventive care, these hypothesis-generating findings make private supply a pragmatic, supply-side reference point for evidence-based revision of the CNAS basket, pointing to two directions directly supported by the data—aligning coverage with observed demand and administrative simplification of contracting. To our knowledge, this is the first benchmarking of the CNAS basket against private supply, relevant to other predominantly private European dental systems.

## 1. Introduction

Oral health is an essential component of general health and well-being, recognized as a *fundamental human right* in the Constitution of the World Health Organization and enshrined in the Universal Declaration of Human Rights [1,2]. Despite this normative recognition, oral diseases remain among the most prevalent non-communicable conditions worldwide, affecting approximately 3.5 billion people and generating substantial individual and social burdens [3]. The adoption of the WHO Resolution on Oral Health in 2021 marked a turning point in international health policy, urging member states to meaningfully integrate oral health into their Universal Health Coverage agendas [4,5]. Nevertheless, the gap between this normative commitment and the financial reality of access to dental care remains substantial. Public sources finance, on average, only one-third of total dental expenditure in Europe, with the remainder borne directly by patients or through voluntary private insurance [6]. This structural gap disproportionately affects low-income and socioeconomically vulnerable populations, producing measurable and persistent inequalities in oral health outcomes across the continent [7,8]. The central question raised by this reality is not solely financial: it concerns the structural capacity of publicly funded dental benefit baskets to address the actual therapeutic needs of the populations they are designed to serve.

Examining this question requires a framework capable of capturing the inherent tensions within health systems. The “*Iron Triangle of Health Care*”—describing the interdependent trade-off between cost, quality, and access—offers precisely such a perspective [9]. Applied to oral health, the model reveals a consistent pattern across European systems: constraints on public dental expenditure compress the scope of covered services, which in turn restricts patient access, ultimately concentrating the burden of unmet needs on those least able to bear it. A comparative analysis of dental coverage across 11 European countries confirmed that national benefit packages are systematically oriented toward curative rather than preventive care, and that substantial disparities in service exclusions and patient co-payments persist across jurisdictions [10]. A broader scoping review of 24 countries established that only six provide comprehensive public dental coverage for adults, while the majority restrict reimbursement to children or emergency care, and that mixed public–private systems disproportionately shift the financial burden onto patients [11]. Crucially, these studies also reveal that the adequacy of any public dental basket cannot be assessed in isolation—it must be evaluated in relation to what patients actually need and seek, not merely in relation to what the system has historically chosen to fund.

Romania illustrates with particular clarity the consequences of this inadequacy. Following its transition from a centralized socialist model to a market-oriented system, Romania underwent a profound structural shift. Whereas in 1999 most dentists practiced in the public sector, by 2005 the private sector dominated, and today roughly 95% of dental services are delivered privately, which is a consequence of chronic underfunding of public dental care, not of deliberate policy [12,13]. For patients, this means that access to dental care is, in practice, conditioned by the ability to pay out of pocket. The Romanian National Health Insurance House (Casa Națională de Asigurări de Sănătate, CNAS) administers the reimbursement of a defined set of dental procedures through framework contracts concluded with accredited providers, covering services at 100% for children and beneficiaries of special laws, and at 60% for complex adult treatments [14,15]. Nevertheless, the monthly reimbursement ceiling per contracted dentist has remained largely stagnant, and the proportion of dental practices operating under a CNAS contract represents a minority of all providers [12]. The result is a system in which the public component is structurally insufficient to absorb demand, geographic distribution is deeply uneven—85.5% of dental practices are concentrated in urban areas, while the rural population faces severely limited access [16]—and the financial burden of oral health falls overwhelmingly on individuals. The Iron Triangle is unbalanced in Romania: cost is shifted to the patient, access is restricted by geography and income, and quality remains unevenly distributed across the territory.

Within this context, a critical analytical gap persists in the literature. Existing studies on oral health in Romania have examined the epidemiological burden, geographic disparities in dentist distribution, socioeconomic barriers to access, and the trajectories of health policy [17,18,19,20]—yet none has addressed a more fundamental question: does the publicly funded benefit package reflect the therapeutic services actively demanded by the patient population? In a system where roughly 95% of dental activity takes place in the private sector, the structure of private service provision offers an indirect, supply-side signal of the therapeutic services patients seek, reflecting what practices choose to offer rather than a directly measured level of demand. This proxy is indirect: it reflects the services practices choose to list, not the services patients are measured to request or receive. Romanian private dental practices do not construct their service portfolios on the basis of administrative logic; they respond to the patients who walk through their doors. What the private market offers therefore provides an indirect, population-level signal of the curative services patients seek that nonetheless remain outside public coverage. This approach may also be transferable to other European systems with a similarly dominant private dental sector and limited voluntary dental insurance.

Against this background, the present study compares the therapeutic service portfolios of 420 private dental practices in Bucharest, Cluj-Napoca, and Iași—three municipalities chosen for their geographic spread—with the CNAS reimbursement basket, and examines how practice size and geographic location shape the breadth of services offered. To our knowledge, this is the first study to benchmark the content of the CNAS basket against the structure of private supply, using the latter as an indirect, supply-side signal of the therapeutic services patients seek rather than as a direct measure of demand. In doing so, it moves beyond describing the structural limitations of the Romanian dental system toward generating evidence that can inform an empirically grounded, demand-aware revision of the public benefit package.

## 2. Materials and Methods

This was a cross-sectional observational study based on publicly available data, collected between November 2024 and February 2025. This study is reported in accordance with the Strengthening the Reporting of Observational Studies in Epidemiology (STROBE) guidelines for cross-sectional studies [21]. A multifaceted analytical approach was applied, combining quantitative analysis of dental service portfolios, organizational data, and the structure of CNAS reimbursement in three major Romanian cities—Bucharest, Cluj-Napoca and Iași. The three municipalities are major university and economic centres located in three different regions of Romania—Bucharest (the national capital and largest market), Cluj-Napoca (north-west), and Iași (north-east). They were selected as the country’s principal urban dental markets, where the private sector is most developed and where price-transparent online data are available, allowing a meaningful public–private comparison.

### 2.1. Data Collection and Unit Identification

Dental practices were identified in two stages. First, practices operating under CAEN code 8623 (dental activities) in the three target municipalities were retrieved from listafirme.ro, a publicly accessible Romanian business-aggregation platform that allows searches by economic-activity code; this provided each practice’s unique fiscal identification number (CUI). Second, the CUI was used to obtain organizational data, including the number of employees and the legal form of organization (SRL—limited liability company). The official website of each practice was then located by searching its registered name on Google and reviewed by a single trained investigator to extract the publicly displayed service portfolio, as this step involved transcription of the listed information rather than clinical judgment. Only practices whose websites publicly listed prices were retained. Because this inclusion criterion depends on online price visibility, it may have preferentially selected larger or more digitally developed practices, an implication we address in the Limitations. The number of practices identified, excluded, and included at each stage is reported in the Results. Price transparency was a prerequisite for valid comparison of service offerings and reflected a level of market maturity and patient-oriented communication consistent with the study’s objectives.

### 2.2. Sample Structure and Stratification

Within the final sample, practices were stratified by number of employees into two categories—small (0–3 employees) and larger (more than 3 employees). Because all eligible price-transparent practices were included rather than a fixed quota per stratum, the strata are unequal in size and reflect the price-transparent segment of practices most visible to patients in each municipality. This stratification enabled cross-group comparison and accounted for the organizational heterogeneity of the Romanian dental market.

### 2.3. Variables and Indicators

The following indicators were used to structure the comparative analysis:

▪ From the CNAS framework contract in force during data collection, we extracted the complete ***list of reimbursed procedures***, their tariffs, and the reimbursement levels by patient category (children 0–18 years; adults over 18 years; beneficiaries of special laws). The 30 procedures were grouped into the five therapeutic categories used by CNAS: basic dental services, endodontic and periodontal services, surgical services, prosthetic services, and orthodontic services.
▪ For each private practice, we then identified which of the 30 CNAS procedures appeared in its published service portfolio. From this coding, we derived two indicators: (i) the ***mean number of CNAS-reimbursed procedures present in private offerings***, by therapeutic category and by municipality; and (ii) the ***proportion of practices not including each individual CNAS procedure***, aggregated into five representation intervals (<25%, 25–49%, 50–74%, 75–99%, and 100%).
▪ The therapeutic services identified in the offerings of private practices were classified into two categories. Functional services were defined as procedures with the primary therapeutic objective of restoring or maintaining oral function and health, including restorative, endodontic, periodontal, surgical, conventional prosthetic, and orthodontic treatments with functional indication. Aesthetic services were defined as procedures whose primary objective is the improvement of the visual appearance of the dentition in the absence of a primary medical indication, including tooth whitening, aesthetic veneers, aesthetic recontouring, and exclusively aesthetic prosthetics. Services were classified on the basis of their primary clinical indication that is, the therapeutic purpose of a procedure rather than the procedure type itself consistent with the way dental benefit baskets in Europe are defined by medical necessity rather than by procedure category [6]. This classification was performed independently by two raters with formal training in dental medicine. Inter-rater agreement for the functional-versus-aesthetic classification, assessed on the consolidated catalogue of 182 procedures (Supplementary Table S1), was almost perfect (observed agreement 99.5%; Cohen’s κ = 0.91, 95% CI 0.72–1.00). CNAS-reimbursed services have exclusively functional character according to the regulations of the framework contract in force.
▪ A ***therapeutic service ratio*** was calculated for each practice as the total number of therapeutic services offered by the practice divided by the number of CNAS-reimbursed services, reflecting how many private services correspond to each publicly funded procedure. Additionally, the percentage difference between the private service portfolio and the CNAS basket was computed to quantify the proportion of privately offered services that fall outside the scope of public reimbursement. The absence of a CNAS-listed procedure from the private offerings of the analyzed practices was interpreted as a possible indicator of limited clinical relevance in current dental practice, although alternative explanations—including equipment requirements, market segmentation, and practice-specific specialization—cannot be excluded. This interpretation rests on the working premise that private practices construct their service portfolios primarily on the basis of expressed demand and clinical judgment, in the absence of administrative obligations to include particular procedures.
▪ Finally, two practice-level characteristics were examined as potential determinants of the breadth of the service portfolio: ***company age***—calculated as the number of years between the establishment date recorded in the registry and the data-collection period—and the number of employees. Each characteristic was related to the total, functional, and aesthetic service counts, both overall and within each municipality and employee-size stratum.

### 2.4. Statistical Analysis

Statistical analysis was performed using IBM SPSS Statistics 25 and Microsoft Office Excel 2024. Continuous variables were expressed as means with standard deviations or medians with interquartile ranges (IQR), depending on distribution. Normality was assessed using the Shapiro–Wilk test. For independent continuous variables with non-parametric distribution, between-group differences were tested using the Kruskal–Wallis H test. Correlations between independent variables with non-parametric distribution were tested using Spearman’s rho correlation coefficients. A significance threshold of α = 0.05 was applied throughout. All comparisons were conducted across the three municipalities and across the two employee size categories, generating a stratified analytical matrix covering six distinct subgroups. In addition, Spearman’s rho correlations were computed between the number of services offered (total, functional, and aesthetic) and two practice characteristics—company age, defined as the number of years from the practice’s establishment date recorded in the registry to the data-collection period, and the number of employees—both overall and within each municipality and employee-size stratum.

Generalized linear models with negative binomial log-link distribution were used to predict the effect of analyzed parameters over the number of offered services. Negative binomial distribution was used in order to avoid overdispersion observed in linear models. Models were tested for significance and goodness-of-fit. Performance of prediction was calculated as incidence rate ratios with 95% confidence intervals along with significance values. Although for all dependent variables, only the number of employees was observed to be a significant predictor, multivariable models were computed in order to account for other possible confounding variables.

## 3. Results

### 3.1. Sample of Analyzed Practices

**Figure 1:**
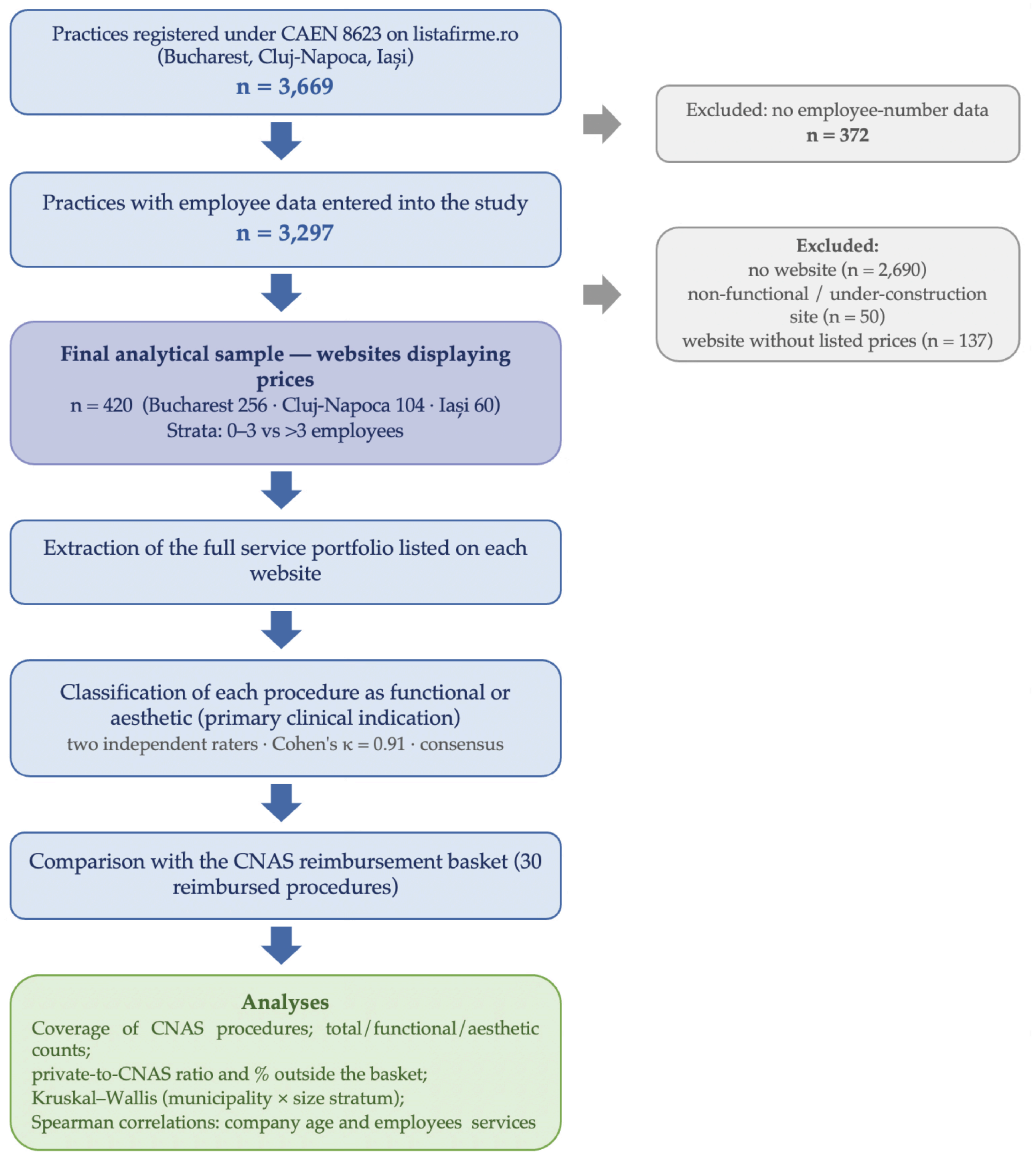
Study workflow, from practice identification and selection through data extraction, service classification, and statistical analysis.

Of the 3669 registered practices, 372 were excluded for lacking employee-number data, leaving 3297; of these, 2690 had no website, 50 had a non-functional or under-construction site, and 137 had a website without listed prices. The final analytical sample comprised 420 practices with publicly listed prices (Bucharest, n = 256; Cluj-Napoca, n = 104; Iași, n = 60). By employee size, this comprised 236 small (0–3 employees) and 184 larger (>3 employees) practices, distributed as follows: Bucharest, 125 small and 131 larger; Cluj-Napoca, 76 small and 28 larger; Iași, 35 small and 25 larger. The selection process is summarized in Figure 1.

### 3.2. The CNAS Basic Dental Services Basket

To enable comparative analysis between private offerings and public funding, Table 1 presents the complete list of dental services reimbursed by CNAS under the framework contract in force during the data collection period, together with the corresponding tariffs and reimbursement levels by patient category. This basket constitutes the reference standard against which the therapeutic portfolios of the analyzed private practices were evaluated in the following sections.

The reimbursed service basket comprises a total of 30 therapeutic procedures, structured across five categories: basic dental services (6 procedures), endodontic and periodontal services (6 procedures), surgical services (6 procedures), prosthetic services (6 procedures), and orthodontic services (6 procedures). Reimbursement is differentiated by patient category: full coverage (100%) for children aged 0–18 years and for beneficiaries of special laws, and partial coverage (60%) for adults over 18 years in the case of complex procedures. Certain services—sealants, fluoride applications, operculectomy, temporomandibular joint dislocation reduction, and orthodontic services with the exception of appliance repair—are available exclusively or predominantly for the pediatric category, with no equivalent in the adult package. Reimbursement frequency for several procedures is also contractually regulated: consultation and dental scaling are reimbursed once per year, sealants once every two years, and removable acrylic dentures once every four years.

Table 2 presents the mean number of CNAS-reimbursed services per therapeutic category, recorded across the analyzed practices in the three municipalities.

According to the data in Table 2, in the basic dental services category, mean values are similar across the three municipalities, ranging from 5.17 (Bucharest) to 5.40 (Cluj-Napoca). In the endodontic and periodontal services category, Iași records the highest mean (3.84), compared with both Bucharest and Cluj-Napoca (3.22 each). In the surgical services category, Iași again shows the highest mean (3.20), versus 2.82 in Bucharest and 2.77 in Cluj-Napoca. In the prosthetic services category, Cluj-Napoca and Iași record similar values (4.41 and 4.38, respectively), compared with 3.28 in Bucharest. In the orthodontic services category, a progressive distribution is observed: 1.29 in Bucharest, 1.74 in Cluj-Napoca, and 2.14 in Iași. Across the five categories, Iași records the highest mean for endodontic and periodontal, surgical, and orthodontic services, while Cluj-Napoca records the highest for basic and prosthetic services (basic being a virtual tie, 5.40 vs. 5.39); Bucharest shows the lowest or near-lowest mean throughout.

### 3.3. Coverage of CNAS-Reimbursed Procedures in the Private Service Portfolios

Table 3 presents the distribution of CNAS-reimbursed dental procedures according to the proportion of private practices in the sample that did not include them in their own offerings, stratified by municipality.

Table 3 summarizes the distribution of the 30 CNAS-reimbursed procedures according to the proportion of private practices that did not include them in their published offerings.

Across all three municipalities, the procedures most consistently present in private offerings (absent from fewer than 25% of practices) are core basic services—consultation, simple caries treatment, dental scaling, and standard extractions. The overall distribution of representation intervals is broadly similar across municipalities, with minor variations only in the 50–74% and 75–99% bands.

At the opposite end of the distribution, the procedures fully absent from every analyzed practice (100%) differ by municipality but consistently belong to specialized surgical and functional orthodontic categories: functional re-education through exercises and myogymnastics in Bucharest; temporomandibular joint dislocation reduction and appliances for the treatment of congenital malformations in Cluj-Napoca; and all three of these procedures in Iași. These near-universally absent procedures share limited clinical applicability in the general urban population, indicating a discrepancy between the current structure of the CNAS basket and the actual profile of expressed therapeutic demand—and providing empirical grounds for reassessing the clinical relevance of certain procedures within the public reimbursement package.

### 3.4. Distribution of Functional and Aesthetic Services in Private Offerings Compared with the CNAS Basket

This section analyzes the gap between the therapeutic offerings of the private sector and the CNAS reimbursement basket from three complementary perspectives: the absolute number of services offered (Table 4), the ratio of private services to the CNAS basket (Table 5), and the percentage of private services falling outside that basket (Table 6). The classification of services as functional or aesthetic follows the operational definitions presented in Section 2.3.

Three principal findings emerge from the comparative analysis presented below. A consistent structural gap exists between the therapeutic offerings of the private sector and the CNAS reimbursement basket. Regardless of municipality and practice size, approximately 53–68% of private therapeutic services fall outside the public reimbursement basket. Contrary to the common perception that private dentistry is dominated by aesthetic procedures, analysis of Table 4 indicates that approximately 95–96% of services offered in the private sector are of a functional nature, with aesthetic services accounting for only 4–5% of the total therapeutic portfolio across all three municipalities. Because the aesthetic component is so small, it cannot account for the magnitude of the gap relative to the CNAS basket: even if aesthetic procedures were entirely excluded from private portfolios, the overwhelming majority of functional services would still remain outside the public reimbursement package. The mismatch between private offerings and the CNAS basket therefore appears to be driven almost entirely by functional services that patients seek and providers deliver, yet which are not reimbursed. No statistically significant differences between municipalities emerged in either size stratum for any analyzed indicator—total, functional, and aesthetic services; private/CNAS ratio; and percentage difference, indicating that geographic location did not produce a decisive divergence in portfolio composition.

It should be noted that the present analysis quantifies the breadth of the offered service portfolios rather than the volume of services delivered; the observed percentages therefore characterize the catalog of services available to patients, not the relative frequency with which each service is consumed.

According to the data in Table 4, no statistically significant differences between municipalities were observed for any service type (*p* > 0.05).

The therapeutic service ratio represents the number of therapeutic services offered by a private practice divided by the number of CNAS-reimbursed services, reflecting how many private services correspond to each publicly funded procedure. According to the data in Table 5, no statistically significant differences between municipalities were observed (*p* > 0.05).

The percentage difference represents the proportion of private therapeutic services that fall outside the CNAS basket, calculated by subtracting the number of reimbursed services from the practice’s total private services and dividing by the original total. According to the data in Table 6, no statistically significant differences between municipalities were observed (*p* > 0.05).

### 3.5. Correlation Between Company Age and the Breadth of the Service Portfolio

Table 7 presents the correlations between company age (years since establishment) and the number of services offered. Across all analyzed subgroups and for every service type (total, functional, and aesthetic) the correlations between company age and the number of services were statistically non-significant (*p* > 0.05), indicating that the breadth of the private service portfolio is not associated with how long a practice has been established.

### 3.6. Correlation Between the Number of Employees and the Breadth of the Service Portfolio

Table 8 presents the correlations between the number of employees and the number of services offered. Among practices with 0–3 employees, no significant correlations were observed in any of the three municipalities (*p* > 0.05). Among practices with more than 3 employees, a significant positive correlation emerged in Bucharest for both total (*p* = 0.009, R = 0.228) and functional services (*p* = 0.009, R = 0.228). When the two strata were pooled within each municipality, significant positive correlations were again confined to Bucharest—total (*p* < 0.001, R = 0.265), functional (*p* < 0.001, R = 0.263), and aesthetic services (*p* = 0.002, R = 0.193). At the national level, practices with more than 3 employees showed significant positive correlations for total (*p* = 0.046, R = 0.148) and functional services (*p* = 0.044, R = 0.149). Across the entire study group, the number of employees correlated significantly with all service types: total (*p* < 0.001, R = 0.217), functional (*p* < 0.001, R = 0.216), and aesthetic (*p* = 0.002, R = 0.149), indicating that practices with more employees tend to offer a larger number of services. All significant correlations were nonetheless weak in magnitude (R values below 0.27).

Data from Table 9 confirms the initial findings, according to the results from the multivariable models, only the existence of more than three employees (vs. 0–3 employees) was a significant and independent predictor for a significant increase of total offered services by 35% (95% C.I. = 10.3–65.2%) (*p* = 0.004), functional offered services by 35.1% (95% C.I. = 10.3–65.4%) (*p* = 0.004) or aesthetic offered services by 32.2% (95% C.I. = 6.3–64.6%) (*p* = 0.012).

## 4. Discussion

### 4.1. Geographic Distribution and the Public–Private Gap

The results of this study suggest that the distribution of dental services in Romania cannot be interpreted solely through the lens of the total number of practices, but must be analyzed simultaneously through the availability of private offerings and the real capacity for public-system access: provider availability is not equivalent to the financial accessibility of treatment, a distinction that becomes essential in a domain where public funding remains limited. A particular case is represented by the larger practices (more than 3 employees) in Bucharest, which simultaneously record the most extensive service portfolio (mean 146.9 services), the highest private/CNAS ratio (4.91), and among the widest percentage differences from the public basket (67.9%)—suggesting that, in the presence of a large and consolidated private market, practices with substantial operational capacity tend to diverge most strongly from the structure of public funding, although these between-municipality differences did not reach statistical significance.

The stratified analysis did not reveal statistically significant geographic specialization. Aesthetic service volume was statistically indistinguishable across municipalities in both strata (*p* = 0.98 for small and *p* = 0.91 for larger practices), and functional service volume likewise showed no significant differences (*p* = 0.087 for small and *p* = 0.26 for larger practices). Organizational scale therefore did not produce the kind of geographic specialization that a more mature market segmentation might predict; across both strata, the three municipalities offered broadly comparable portfolio profiles.

This interpretation aligns with recent European literature, which shows that dentistry remains among the most poorly covered sectors by public health schemes, with substantial differences between countries in terms of financing, access, and the degree of financial protection for adults. At the European level, public coverage of adult dental services is highly variable, and unmet needs are greater for dental care than for other medical services, disproportionately affecting low-income groups [21]. For Romania, the OECD/European Observatory profile explicitly shows that dental care is almost entirely privately financed; in 2021, only 5% of dental expenditure was publicly funded, compared with an EU average of 34% [12]. Eurostat data further indicate that, in 2024, Romania was among the countries with the highest proportions of unmet dental needs, with a particularly wide gap between persons at risk of poverty and those not at risk [22,23]. Our results must therefore be interpreted in a broader context in which the private market absorbs the majority of demand, while the public system remains a limited mechanism of protection for vulnerable groups.

### 4.2. Adequacy of the CNAS Basket Relative to Expressed Therapeutic Demand

Before interpreting this gap, an important caveat must be restated, the presence of a service in a practice’s online portfolio indicates that the clinic is willing and equipped to provide it, not how often patients request it, how frequently it is delivered, or whether it is offered because it is clinically needed rather than commercially attractive. Throughout, private supply is therefore treated as an indirect, supply-side signal, not a direct measure of demand.

A second major result of this study is the discrepancy between the list of CNAS-reimbursable services and the actual offerings of private practices. Approximately 53% to 68% of private therapeutic services fall outside the public reimbursement basket, regardless of municipality or practice size. An essential observation in interpreting this gap concerns its composition: approximately 95–96% of services offered in the private sector are functional in nature, while aesthetic services account for only 4–5% of the total therapeutic portfolio. The aesthetic share is therefore far too small to account for a gap of this magnitude relative to the CNAS basket; even if aesthetic procedures were excluded entirely from the comparison, the great majority of functional services would still fall outside public reimbursement. Contrary to the common perception that private dentistry is dominated by aesthetic procedures, the discrepancy with the CNAS basket is therefore driven almost entirely by functional services providers offer, yet which remain absent from the public reimbursement package. This finding reframes the nature of the reform argument: the problem is not that CNAS fails to cover aesthetic services—this is expected and justified—but that it fails to cover a significant portion of expressed functional demand.

The fact that numerous CNAS procedures are poorly represented or absent from private offerings suggests not only a difference in clinical orientation, but also a problem of economic and organizational attractiveness of contracting. Importantly, such absences need not have a single interpretation: they may reflect provider preferences and specialization, the equipment or training a procedure requires, or its commercial attractiveness and market positioning; these considerations weigh most heavily on the more complex or resource-intensive interventions, which are correspondingly less likely to appear in the routine portfolios of general private practice. In health-economics terms, the private sector primarily responds to solvent demand and only indirectly to total medical need within the population. For this reason, an important methodological distinction is required: the present study uses private supply as an indicator of expressed demand translated into actual medical consumption, not as a measure of total epidemiological need—the two concepts are related but not identical. Expressed demand reflects what patients seek and consume under current conditions of access and affordability, while total epidemiological need also encompasses cases in which clinical necessity exists but does not translate into actual utilization due to financial or geographic barriers.

The theoretical framework requires an acknowledgement of its limitations, as health economics identifies three critical perspectives on the supply–demand model in the medical domain. Information asymmetry means the patient cannot fully evaluate their own clinical need and depends on the provider’s judgment, which can distort expressed demand relative to objective need [24]. Supplier-induced demand means that, in absent robust regulation, providers may orient supply toward services with higher economic margins, thereby shaping demand rather than reflecting it [25]. The third, least discussed in the Romanian literature, refers to the cultural modeling of demand: beliefs about health, traditional patterns of medical consumption, and health-literacy levels act as filters that determine which symptoms are perceived as medical problems and when they translate into actual provider visits [26].

In the Romanian context, this third perspective takes a particular form: a predominant orientation toward curative care and a systematic underutilization of prevention. Eurostat data place Romania among the countries with the lowest rates of preventive dental visits in the European Union [22,23], and Caramida et al. confirm, from the perspective of Romanian patients, a reactive utilization pattern triggered by pain or dysfunction rather than proactive prevention [19]. This cultural configuration introduces a predictable methodological asymmetry: private supply faithfully captures expressed curative demand but structurally under-represents unexpressed preventive need. Rather than weakening the central argument of the study, this observation sharpens it. The CNAS basket is itself largely a curative inventory, with only limited exceptions in the preventive area (sealants, fluoride applications, dental scaling). The private—CNAS comparison underlying our results is therefore a comparison between two inventories aligned on the same reactive logic, and the observed gap reflects a real mismatch within the curative domain, not an artifact of the difference between expressed demand and total epidemiological need.

Despite these caveats, private supply remains a pragmatic and valuable signal for evaluating the adequacy of the public basket—particularly in a system where over 95% of dental activity occurs in the private sector, without administrative constraints on portfolio composition. The international literature supports this interpretation: in systems where private-provider participation in public programs depends on remuneration levels and administrative burden, contracting tends to be limited when tariffs are perceived as below market or when bureaucratic requirements are excessive. Studies from the United States on Medicaid programs have shown that fee increases and administrative reforms can raise dentist participation and service utilization, while barriers such as low reimbursement, complex procedures, and insufficient institutional support reduce provider engagement [27,28]. Similarly, the British literature has described the shift of a portion of dental care toward the private sector primarily as an issue of access and contractual organization, not solely as an expression of patient preferences [29,30]. In England, public-system orthodontic treatment for adults is typically restricted to cases of severe or complex need, confirming that many public systems strictly delimit the functional from the aesthetic domain [29].

It is important to mention that a divergence between public coverage and private market offerings is expected as public reimbursement systems are deliberately designed to prioritize essential and cost-effective interventions, not to reproduce the full breadth of services available in the market. The 53–68% of listed private services that fall outside the CNAS basket therefore identifies areas of potential misalignment that merit closer examination, particularly where the excluded services are functional treatments routinely sought by patients.

### 4.3. Organizational Size and Practice Longevity as Differentiating Factors

Across both size strata, differences between municipalities were statistically non-significant for all analyzed indicators—total, functional, and aesthetic services; private/CNAS ratio; and percentage difference. Geographic location therefore did not act as a decisive differentiator of the service portfolio at either level of operational capacity, and the data do not support the notion that portfolio divergence is driven primarily by larger practices. In policy terms, this suggests that the structural gap between private supply and the CNAS basket is a system-wide feature rather than one concentrated in a particular size segment, so that contracting reforms need to address the full range of practice sizes rather than targeting larger units alone.

Two practice-level characteristics were examined as possible determinants of this portfolio breadth. The first, company age, showed no significant association with the number of services in any subgroup (Section 3.6). The longevity of a practice therefore does not translate into a broader service catalogue: more recently established practices offer portfolios comparable to those of long-standing ones. This is consistent with the interpretation that private portfolios are shaped by current market positioning and expressed patient demand rather than by the gradual accumulation of services over time.

The second characteristic, the number of employees, was the practice attribute most consistently associated with portfolio breadth. It correlated significantly with the number of services across the full sample (total *p* < 0.001; Section 3.6) and, in the multivariable generalized linear models, was the only significant independent predictor: practices with more than three employees offered approximately 33% more services (total +35%, functional +35%, aesthetic +32%) after adjustment for municipality and company age, neither of which was a significant predictor (Table 9). Greater staffing capacity is thus independently associated with a broader service offering a plausible reflection of the additional clinical and logistical capacity that more personnel provide. This size effect concerns the breadth (number) of services rather than the functional–aesthetic composition or the magnitude of the gap relative to the CNAS basket, which remained system-wide. The European literature notes that dental care provision is increasingly organized in larger structures, making the analysis of organizational size ever more relevant for contracting and regulatory policies [6].

### 4.4. International Comparative Perspective

A recent analysis classifying all 194 WHO Member States identified four distinct models of oral-health integration within public-financing mechanisms: complete structural integration into UHC, partial or targeted integration, predominantly private or insurance-driven systems, and minimal or emerging integration. According to this typology, only 10% of states have achieved full structural integration, while 17%—the category in which Romania falls—operate as predominantly private or insurance-driven systems [5]. This positioning provides a useful framework for interpreting our results, placing the Romanian dental system within a minority but consistent group characterized by the majority transfer of care to the private sector.

Comparison with other countries is relevant both from the perspective of market organization and that of public-funding limits. In Canada, dental care has long been situated outside the core of the universal system, and a recent national report indicates that only 5–6% of dental services were paid for from public funds, with the remainder covered through private insurance or out-of-pocket payments [31,32]. In New Zealand, adult dental services are largely user-pays, and health-geography studies have shown that private practices tend to concentrate in areas of higher socioeconomic status rather than in areas with the greatest disease burden [33]. In Australia, access to public services has been associated with persistent waiting lists [34,35]. Some analyses have proposed extending access by subcontracting public services to participating private practices, demonstrating that this approach could improve geographic access particularly in rural areas [36]. Our findings regarding the ratio of functional to aesthetic services reinforce the idea that two distinct operational logics coexist in all these systems. The CNAS basket is structured almost exclusively around functional restoration and basic interventions, whereas the private sector includes a much broader range of services. This difference is not unique to Romania—most European public systems prioritize children, vulnerable groups, and treatments deemed medically necessary, while elective services remain outside public coverage [6,13]. The problem is not the existence of this prioritization, but the magnitude of the gap between the public package and actual market supply, which may amplify the segmentation of access between patients who can afford complex treatments privately and those dependent on the public package. These examples show that Romania is not an isolated case, but rather falls within a pattern observed in systems where dentistry is heavily dependent on the private market.

### 4.5. Healthcare Policy Implications

The international experiences described above confirm that Romania is not a singular case, but rather falls within a structural pattern shared by systems with high private-sector dependence in dentistry. They also provide a useful frame of reference for formulating public-policy recommendations adapted to the local context, in which contracting mechanisms and the structure of the public basket represent the principal available levers for intervention.

Given the cross-sectional, observational design of this study, the directions outlined below are offered as hypotheses to inform future evaluation rather than as direct policy prescriptions. From a public-policy standpoint, the results of this study may support the need for a periodic revision of the CNAS package based on three criteria: population need, actual service utilization, and the attractiveness of contracting for providers. A first direction might be to update the list of services and tariffs so that the public package better reflects contemporary clinical practice. This requires both the recalibration of existing entries through the reassessment of CNAS-listed procedures whose representation in current private offerings has diminished, and the consideration of new inclusions corresponding to functional services routinely sought by patients in the private market but currently outside public coverage. A second direction might be administrative simplification and the creation of meaningful incentives for private-practice participation. Because the gap from the public basket is a system-wide feature rather than one concentrated in a particular size segment (Section 4.3), these measures should address the full range of practices rather than any single size category. International experience suggests that the mere existence of a public package is insufficient; without adequate tariffs, feasible contractual rules, and mechanisms for integrating private providers, access remains unequal [27,35,36].

**Figure 2:**
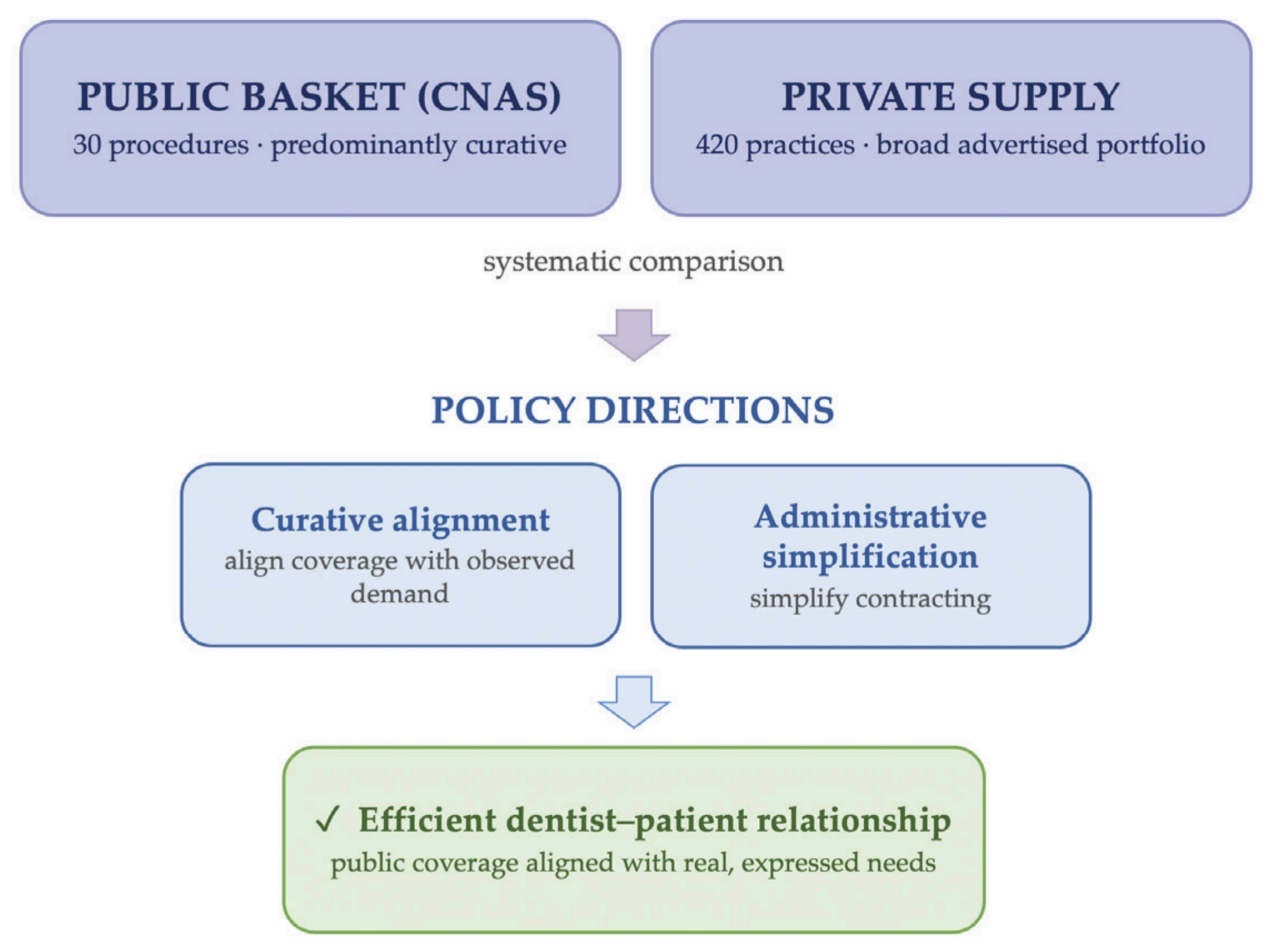
Conceptual framework synthesizing the analytical logic and policy implications of the present study.

These two directions might form an integrated conceptual framework for reform (Figure 2), connecting the methodological comparison between private supply and the CNAS basket to the ultimate objective: an efficient dentist-patient relationship capable of meeting the actively expressed therapeutic demand of the population.

### 4.6. Strengths and Limitations

The strengths of the study include the simultaneous analysis of three county-seat municipalities, the stratification of practices by organizational size, and the direct comparison between publicly reimbursable services and the declared offerings of the private market on a sample of 420 practices. To our knowledge, this is also the first study to benchmark the CNAS reimbursement basket against the structure of private dental supply.

The results, however, must be interpreted in light of several limitations. First, the analysis is based on the offerings declared on practice websites and characterizes the breadth of available service portfolios rather than the actual volume of services delivered or reported to CNAS; the observed percentages therefore reflect the catalog of services available, not the relative frequency with which patients consume each service. Second, the study has a cross-sectional design and captures a snapshot of the market within a limited time interval. Third, the three analyzed municipalities are major university and economic centers, such that the results cannot be automatically extrapolated to smaller cities or to rural areas, where access barriers may differ and are likely more severe. Finally, the consecutive sampling strategy based on Google search results favored practices with established online presence, strong search-engine visibility, and explicit price transparency, which may have over-represented commercially and digitally mature practices; the findings should therefore be interpreted as descriptive of the digitally visible segment of the Romanian private dental market, rather than of the full universe of practices. In quantitative terms, the final sample represented approximately 11% of all registered practices; this low inclusion proportion reflects the requirement for a publicly accessible, price-listing website rather than survey non-response, since only a minority of practices publish prices online

A comprehensive understanding of the dimensions of the private—CNAS relationship would also benefit from a complementary analysis of the tariff structure of dental services. While the present study has focused on the breadth of services offered, the question of how those services are priced—relative to public reimbursement levels—represents an important direction for future research.

## 5. Conclusions

This study indicates that, in Romania, a consistent structural gap exists between the therapeutic offerings of the private dental sector and the CNAS reimbursement basket, with 53–68% of private services remaining outside public funding. Contrary to common perception, this appears not to be explained by aesthetic services (which account for only 4–5% of private portfolios, while approximately 95–96% are functional) but rather by the existence of functional services routinely offered in private practice, yet which remain absent from the public reimbursement package. Geographic location was not associated with portfolio breadth, whereas larger practices (>3 employees) offered significantly more services; nonetheless, the structural gap between private supply and the CNAS basket (the proportion of services left unfunded) remained a system-wide feature, not one concentrated in a particular municipality or practice-size segment. The central issue is therefore not the insufficiency of public financing alone, but the mismatch between the architecture of the CNAS basket and the actual operation of the private market.

These results are best regarded as hypothesis-generating. Because private service availability is shaped not only by patient demand but also by provider preferences, profitability, equipment, and local market factors—and because the study measures neither treatment utilization, epidemiological need, nor cost-effectiveness—its findings should be read as raising questions about the alignment between public coverage and private market supply rather than as definitive evidence that the reimbursement basket should be restructured. Moreover, as the analysis was confined to the price-transparent, digitally visible practices of three major urban markets, these observations should be interpreted within that scope rather than generalized to smaller towns or rural areas, and they identify priorities for the prospective, utilization- and needs-based evaluations required before any revision of the CNAS package.

## Figures and Tables

**Table 1:** CNAS-reimbursed dental services, corresponding tariffs, and reimbursement levels by patient category.

No.	Therapeutic Procedure	Tariff (RON)	Amount Reimbursed by CNAS			Service Category
			Children 0–18 yrs	Adults >18 yrs	Beneficiaries of Special Laws	
1	Consultation—including oral oncology screening, plaque disclosure (where applicable), and treatment plan	149	100%	100%	100%	***Basic dental services***
2	Treatment of simple caries	157	100%	100%	100%	
3	Tooth restoration following pulp pathology or gangrene treatment	207	100%	60%	100%	
4	Sealant per tooth	108	100%	-	-	
5	Fluoride application (per dental arch)	94	100%	-	-	
6	Ultrasonic dental scaling and professional polishing	150	100%	100%	100%	
7	Treatment of pulp pathology with anesthesia	269	100%	60%	100%	***Endodontic and periodontal*** ***services***
8	Sedative dressing/endodontic drainage	78	100%	100%	100%	
9	Treatment of pulp gangrene	314	100%	60%	100%	
10	Treatment of apical periodontitis—by incision—with anesthesia	190	100%	100%	100%	
11	Treatment of periodontal conditions with anesthesia	131	100%	100%	100%	
12	Treatment of oral mucosa conditions	76	100%	60%	100%	
13	Emergency treatment of dento-alveolar trauma, per tooth	196	100%	100%	100%	***Surgical*** ***services***
14	Extraction of primary teeth with anesthesia	76	100%	60%	100%	
15	Extraction of permanent teeth with anesthesia	162	100%	60%	100%	
16	Alveolar curettage and treatment of bleeding	112	100%	100%	100%	
17	Operculectomy	87	100%	-	-	
18	Reduction in temporomandibular joint dislocation	86	100%	100%	100%	
19	Removable acrylic denture per arch	1145	-	100%	100%	***Prosthetic*** ***services***
20	Denture repair	138	-	100%	100%	
21	Denture relining	202	-	100%	100%	
22	Aesthetic prosthetic element	224	100%	60%	100%	
23	Semi-aesthetic prosthetic element	258	100%	60%	100%	
24	Post-and-core restoration	263	100%	60%	100%	
25	Functional habit-breaking via orthodontic appliances, including treatment of crossbite with bands/splints, chincup, and headcap	866	100%	-	-	***Orthodontic services***
26	Functional re-education through exercises and myogymnastics, per session	22	100%	-	-	
27	Appliances and devices used in the treatment of congenital malformations	1094	100%	-	-	
28	Orthodontic interproximal stripping, per tooth	28	100%	-	-	
29	Repair of orthodontic appliance	583	100%	100%	-	
30	Removable space maintainers	656	100%	-	-	

**Table 2:** Mean number of CNAS-reimbursed dental services per therapeutic category, by analyzed municipality.

Service Category	No. of Reimbursed Services	Bucharest	Cluj-Napoca	Iași
Basic dental services	6	Mean: 5.17	Mean: 5.40	Mean: 5.39
Endodontic and periodontal services	6	Mean: 3.22	Mean: 3.22	Mean: 3.84
Surgical services	6	Mean: 2.82	Mean: 2.77	Mean: 3.20
Prosthetic services	6	Mean: 3.28	Mean: 4.41	Mean: 4.38
Orthodontic services	6	Mean: 1.29	Mean: 1.74	Mean: 2.14

**Table 3:** Distribution of CNAS-reimbursed dental procedures according to the proportion of private practices that did not include them in their own offerings, by municipality.

*% of Practices not Including CNAS-Reimbursable Procedures in Private Offering*	*Bucharest*	*Cluj-Napoca*	*Iași*
100%	1	2	3
75%–99%	6	0	6
50–74%	4	5	2
25–49%	8	8	9
<25%	11	15	10

**Table 4:** Comparison of the number of total, functional, and aesthetic therapeutic services by municipality and number of employees.

***Practices with 0–3 employees***				
**Municipality/Services**		**Total**	**Functional**	**Aesthetic**
**Bucharest**	**Mean** ± **SD**	104.64 ± 56.75	100.37 ± 54.95	4.27 ± 2.8
	**95% C.I.**	94.59–114.69	90.64–110.1	3.78–4.77
	**Median (IQR)**	98 (63–137.5)	94 (60–133)	4 (2–6)
**Cluj-Napoca**	**Mean** ± **SD**	114.57 ± 75.64	110.21 ± 73.79	4.36 ± 3.16
	**95% C.I.**	97.28–131.85	93.35–127.07	3.63–5.08
	**Median (IQR)**	100 (51.25–174.25)	95.5 (46.75–167)	4 (2–6.75)
**Iași**	**Mean** ± **SD**	84.37 ± 56.68	80.2 ± 54.97	4.17 ± 3.14
	**95% C.I.**	64.9–103.84	61.32–99.08	3.09–5.25
	**Median (IQR)**	76 (35–111)	70 (32–107)	4 (2–6)
***p***		0.095 *	0.087 *	0.981 *
***Practices with >3 employees***				
**Municipality/Services**		**Total**	**Functional**	**Aesthetic**
**Bucharest**	**Mean** ± **SD**	146.91 ± 92.63	141.51 ± 90.3	5.4 ± 3.76
	**95% C.I.**	130.9–162.92	125.9–157.12	4.75–6.05
	**Median (IQR)**	131 (78–203)	126 (74–197)	5 (2–7)
**Cluj-Napoca**	**Mean** ± **SD**	124.36 ± 84.09	117.75 ± 78.31	6.61 ± 8.32
	**95% C.I.**	91.75–156.97	87.38–148.12	3.38–9.83
	**Median (IQR)**	105.5 (58.5–184)	103 (55.25–174)	4 (2.25–7)
**Iași**	**Mean** ± **SD**	116.16 ± 63.25	111.24 ± 61.78	4.92 ± 2.64
	**95% C.I.**	90.05–142.27	85.74–136.74	3.83–6.01
	**Median (IQR)**	102 (71–166.5)	98 (68.5–159.5)	5 (3–7.5)
***p***		0.265 *	0.255 *	0.914 *

* *Kruskal–Wallis H Test, SD = Standard deviation, IQR = Interquartile range, 95% C.I. = 95% Confidence intervals.*

**Table 5:** Comparison of the ratio of private therapeutic services to the CNAS basket, by municipality and number of employees.

***Practices with 0–3 employees***			
**Municipality/Services**		**Total**	**Functional**
**Bucharest**	**Mean** ± **SD**	3.53 ± 1.82	3.38 ± 1.77
	**95% C.I.**	3.215–3.860	3.068–3.696
	**Median (IQR)**	3.26 (2.1–4.58)	3.13 (2–4.43)
**Cluj-Napoca**	**Mean** ± **SD**	3.87 ± 2.45	3.72 ± 2.4
	**95% C.I.**	3.310–4.432	3.173–4.270
	**Median (IQR)**	3.33 (1.7–5.8)	3.18 (1.55–5.56)
**Iași**	**Mean** ± **SD**	2.97 ± 1.77	2.76 ± 1.72
	**95% C.I.**	2.362–3.579	2.172–3.357
	**Median (IQR)**	2.6 (1.7–3.8)	2.33 (1.06–3.56)
***p***		0.126 *	0.083 *
***Practices with >3 employees***			
**Municipality/Services**		**Total**	**Functional**
**Bucharest**	**Mean** ± **SD**	4.91 ± 3.05	4.73 ± 2.98
	**95% C.I.**	4.391–5.448	4.222–5.253
	**Median (IQR)**	4.36 (2.6–6.76)	4.2 (2.46–6.56)
**Cluj-Napoca**	**Mean** ± **SD**	4.22 ± 2.69	4 ± 2.5
	**95% C.I.**	3.182–5.272	3.032–4.975
	**Median (IQR)**	3.51 (1.95–6.13)	3.43 (1.84–5.8)
**Iași**	**Mean** ± **SD**	3.93 ± 2.02	3.74 ± 2
	**95% C.I.**	3.094–4.766	2.911–4.569
	**Median (IQR)**	3.4 (2.36–5.55)	3.26 (2.28–5.31)
***p***		0.309 *	0.260 *

* *Kruskal–Wallis H Test, SD = Standard deviation, IQR = Interquartile range, 95% C.I. = 95% Confidence intervals.*

**Table 6:** Comparison of the percentage difference between private therapeutic services and the CNAS basket, by municipality and number of employees.

***Practices with 0–3 employees***			
**Municipality/Services**		**% Difference (Total)**	**% Difference Functional**
**Bucharest**	**Mean** ± **SD**	62.91 ± 20.72	60.2 ± 23.94
	**95% C.I.**	59.24–66.58	55.96–64.44
	**Median (IQR)**	69.38 (52.36–78.18)	68.08 (49.98–77.44)
**Cluj-Napoca**	**Mean** ± **SD**	59.4 ± 28	56.95 ± 30.29
	**95% C.I.**	53.01–60.87	50.02–63.87
	**Median (IQR)**	69.92 (41.45–82.75)	68.51 (35.77–81.99)
**Iași**	**Mean** ± **SD**	52.55 ± 28.95	48.49 ± 30.58
	**95% C.I.**	42.61–62.50	37.98–59
	**Median (IQR)**	61.53 (41.17–73.68)	57.14 (6.25–71.96)
***p*** *		0.126	0.083
***Practices with >3 employees***			
**Municipality/Services**		**% Difference (Total)**	**% Difference Functional**
**Bucharest**	**Mean** ± **SD**	67.88 ± 24.57	66.36 ± 25.8
	**95% C.I.**	63.63–72.13	61.9–70.82
	**Median (IQR)**	77.1 (61.53–85.22)	76.19 (59.45–84.77)
**Cluj-Napoca**	**Mean** ± **SD**	62.06 ± 29.22	60.21 ± 30.56
	**95% C.I.**	50.72–73.39	48.35–72.06
	**Median (IQR)**	71.55 (47.11–83.68)	70.85 (43.94–82.68)
**Iași**	**Mean** ± **SD**	67.57 ± 15.92	64.31 ± 20.84
	**95% C.I.**	61–74.14	55.7–72.91
	**Median (IQR)**	70.58 (57.53–81.97)	69.38 (55.91–81.18)
***p*** *		0.309	0.260

* *Kruskal–Wallis H Test, SD = Standard deviation, IQR = Interquartile range, 95% C.I. = 95% Confidence intervals.*

**Table 7:** Correlations between company age and the number of services offered, by municipality and number of employees (Spearman’s rho).

***Practices with 0–3 employees***			
**Total**	**Bucharest**	**Cluj-Napoca**	**Iași**
Correlation (*p* *)	0.436, R = −0.070	0.407, R = 0.097	0.591, R = 0.094
**Functional**	**Bucharest**	**Cluj-Napoca**	**Iași**
Correlation (*p* *)	0.459, R = −0.067	0.387, R = 0.101	0.559, R = 0.102
**Aesthetic**	**Bucharest**	**Cluj-Napoca**	**Iași**
Correlation (*p* *)	0.203, R = −0.115	0.297, R = 0.121	0.930, R = −0.015
***Practices with >3 employees***			
**Total**	**Bucharest**	**Cluj-Napoca**	**Iași**
Correlation (*p* *)	0.175, R = −0.119	0.445, R = −0.150	0.291, R = −0.220
**Functional**	**Bucharest**	**Cluj-Napoca**	**Iași**
Correlation (*p* *)	0.179, R = −0.118	0.456, R = −0.147	0.227, R = −0.251
**Aesthetic**	**Bucharest**	**Cluj-Napoca**	**Iași**
Correlation (*p* *)	0.429, R = −0.070	0.446, R = −0.150	0.951, R = 0.013
***Cumulated groups***			
**Total**	**Bucharest**	**Cluj-Napoca**	**Iași**
Correlation (*p* *)	0.283, R = −0.067	0.734, R = 0.034	0.698, R = 0.051
**Functional**	**Bucharest**	**Cluj-Napoca**	**Iași**
Correlation (*p* *)	0.282, R = −0.067	0.723, R = 0.035	0.717, R = 0.048
**Aesthetic**	**Bucharest**	**Cluj-Napoca**	**Iași**
Correlation (*p* *)	0.229, R = −0.075	0.586, R = 0.054	0.879, R = 0.020
***National level***			
Correlation (*p* *)	**Total**	**Functional**	**Aesthetic**
**0–3 employees**	0.760, R = 0.020	0.726, R = 0.023	0.783, R = −0.018
**>3 employees**	0.200, R = −0.095	0.195, R = −0.096	0.477, R = −0.053
**Entire study group**	0.927, R = 0.004	0.936, R = 0.004	0.737, R = −0.016

* *Spearman’s rho correlation coefficient.*

**Table 8:** Correlations between the number of employees and the number of services offered, by municipality and number of employees (Spearman’s rho).

***Practices with 0–3 employees***			
**Total**	**Bucharest**	**Cluj-Napoca**	**Iași**
Correlation (*p* *)	0.360, R = 0.083	0.351, R = 0.108	0.639, R = 0.082
**Functional**	**Bucharest**	**Cluj-Napoca**	**Iași**
Correlation (*p* *)	0.371, R = 0.081	0.341, R = 0.111	0.664, R = 0.076
**Aesthetic**	**Bucharest**	**Cluj-Napoca**	**Iași**
Correlation (*p* *)	0.111, R = 0.143	0.765, R = 0.035	0.367, R = −0.157
***Practices with >3 employees***			
**Total**	**Bucharest**	**Cluj-Napoca**	**Iași**
Correlation (*p* *)	**0.009, R = 0.228**	0.710, R = −0.073	0.620, R = −0.104
**Functional**	**Bucharest**	**Cluj-Napoca**	**Iași**
Correlation (*p* *)	**0.009, R = 0.228**	0.742, R = −0.065	0.612, R = −0.107
**Aesthetic**	**Bucharest**	**Cluj-Napoca**	**Iași**
Correlation (*p* *)	0.075, R = 0.156	0.212, R = −0.244	0.953, R = −0.012
***Cumulated groups***			
**Total**	**Bucharest**	**Cluj-Napoca**	**Iași**
Correlation (*p* *)	**<0.001, R = 0.265**	0.407, R = 0.082	0.062, R = 0.242
**Functional**	**Bucharest**	**Cluj-Napoca**	**Iași**
Correlation (*p* *)	**<0.001, R = 0.263**	0.402, R = 0.083	0.064, R = 0.241
**Aesthetic**	**Bucharest**	**Cluj-Napoca**	**Iași**
Correlation (*p* *)	**0.002, R = 0.193**	0.570, R = 0.056	0.594, R = 0.070
***National level***			
Correlation (*p* *)	**Total**	**Functional**	**Aesthetic**
**0–3 employees**	0.157, R = 0.092	0.153, R = 0.093	0.285, R = 0.070
**>3 employees**	**0.046, R = 0.148**	**0.044, R = 0.149**	0.230, R = 0.089
**Entire study group**	**<0.001, R = 0.217**	**<0.001, R = 0.216**	**0.002, R = 0.149**

* *Spearman’s rho correlation coefficient.*

**Table 9:** Univariable and multivariable generalized linear models (negative binomial log-link distribution) used in the prediction of the number of offered services.

No. Offered Services (Total)	Univariable		Multivariable	
	IRR (95% C.I.)	*p*	IRR (95% C.I.)	*p*
**Municipality**				
**Bucharest (Ref.)**	**-**	**-**	**-**	**-**
**Iași**	0.928 (0.738–1.167)	0.928	0.990 (0.779–1.260)	0.937
**Cluj-Napoca**	0.773 (0.583–1.025)	0.074	0.769 (0.574–1.030)	0.078
**Company age (years)**	1.000 (0.988–1.012)	0.990	0.994 (0.982–1.007)	0.357
**No. employees**	1.015 (1.003–1.026)	0.010	-	-
**No. employees > 3**	1.329 (1.095–1.613)	0.004	1.350 (1.103–1.652)	0.004
**No. offered services (Functional)**	**Univariable**		**Multivariable**	
	**IRR (95% C.I.)**	***p***	**IRR (95% C.I.)**	***p***
**Municipality**				
**Bucharest (Ref.)**	**-**	**-**	**-**	**-**
**Iași**	0.924 (0.735–1.162)	0.501	0.988 (0.776–1.256)	0.919
**Cluj-Napoca**	0.767 (0.578–1.017)	0.066	0.763 (0.570–1.022)	0.070
**Company age (years)**	1.000 (0.988–1.012)	0.980	0.994 (0.982–1.007)	0.357
**No. employees**	1.015 (1.004–1.026)	0.009	-	-
**No. employees > 3**	1.331 (1.096–1.615)	0.004	1.351 (1.103–1.654)	0.004
**No. offered services (Aesthetic)**	**Univariable**		**Multivariable**	
	**IRR (95% C.I.)**	***p***	**IRR (95% C.I.)**	***p***
**Municipality**				
**Bucharest (Ref.)**	-	**-**	-	**-**
**Iași**	1.023 (0.797–1.314)	0.856	1.061 (0.818–1.375)	0.657
**Cluj-Napoca**	0.925 (0.678–1.262)	0.622	0.922 (0.668–1.271)	0.619
**Company age (years)**	0.998 (0.985–1.011)	0.780	0.994 (0.980–1.008)	0.420
**No. employees**	1.006 (0.993–1.018)	0.367	-	-
**No. employees > 3**	1.288 (1.042–1.591)	0.019	1.322 (1.063–1.646)	0.012

## Data Availability

The original contributions presented in this study are included in the article. Further inquiries can be directed to the corresponding author.

## Supplementary Materials

The following supporting information can be downloaded at: https://www.mdpi.com/article/10.3390/healthcare14152349/s1, Supplementary Table S1. Full classification list of dental procedures.
