# Supplementary material for: Misalignment Between Public Reimbursement and Private Dental Service Provision in Romania: A Cross-Sectional Analysis of 420 Dental Practices in Three Major Municipalities
Source: Healthcare (Basel). 2026 Aug 1;14(15):2349. doi: 10.3390/healthcare14152349 (PMC13465268; doi:10.3390/healthcare14152349)
Supplement: Supplementary file 1 [file healthcare-14-02349-s001.zip › healthcare-4426660-supplementary.pdf]

Supplementary Table S1. Full classification list of dental procedures

Consolidated catalogue of the 182 distinct procedures identified in the publicly listed portfolios of the 420 analyzed private dental practices, classified as functional or aesthetic according to primary clinical indication. Classification was performed independently by two trained raters (observed agreement 99.5%; Cohen's κ = 0.91); the single discrepancy was resolved by consensus.

No.	Procedure (English)	Original term (Romanian)	Classification
1	Removal of prosthetic restoration	Ablație lucrare protetică	Functional
2	Endodontic access / trephination	Acces / Trepanare endodontică	Functional
3	Acclimatisation visit (for children or patients with dental anxiety)	Acomodare	Functional
4	Vestibular sulcus deepening	Adancire sant vestibular	Functional
5	Splint adaptation	Adaptare splint	Functional
6	Bone augmentation / guided bone regeneration (GBR)	Adiție osoasă / GBR	Functional
7	Air polishing (air-flow)	Air-flow	Functional
8	Tooth whitening (bleaching)	Albire dentară	Aesthetic
9	Aligners / orthodontic trays	Aligneri / Gutiere ortodontice	Functional
10	Surgical crown lengthening	Alungire / elongare coronară (chirurgical)	Functional
11	Alveoloplasty / ridge recontouring	Alveoloplastie / Regularizare creastă	Functional
12	Impression / intraoral scanning	Amprentă / Scanare intraorală	Functional
13	Root amputation / hemisection	Amputație / Hemisecție radiculară	Functional
14	Anaesthesia	Anestezie	Functional
15	Nance appliance / transpalatal arch	Aparat Nance / Bară transpalatinală	Functional
16	Orthodontic appliance	Aparat ortodontic	Functional
17	Partial archwire placement	Aplicare arc partial	Functional
18	Periodontal dressing application	Aplicare ciment parodontal	Functional
19	Devitalizing paste application	Aplicare devitalizant (pastă)	Functional
20	Mineral trioxide aggregate (MTA) application	Aplicare MTA	Functional
21	Plaque-disclosing agent application	Aplicare revelator placa	Functional
22	Topical fluoride application	Aplicatie topica fluor	Functional
23	Lingual arch / bar	Arc / Bară linguală	Functional
24	Glass-ionomer base	Baza glassionomer	Functional
25	Tooth jewellery	Bijuterie dentară	Aesthetic
26	Prosthetic abutment	Bont protetic (abutment)	Functional
27	Healing abutment	Bont vindecare	Functional
28	Protective splints (trays)	Cape protectie	Functional
29	Cauterization	Cauterizare	Functional
30	CBCT / tomography	CBCT / Tomografie	Functional
31	Mucogingival surgery (graft / recession)	Chirurgie mucogingivală (grefă/recesiune)	Functional
32	Cystectomy	Chistectomie	Functional
33	Cementation / re-cementation	Cimentare / Recimentare	Functional
34	Pulp capping	Coafaj pulpar	Functional
35	Bonding of metal button	Colare buton metalic	Functional
36	Consultation / examination	Consultație / Examinare	Functional
37	Retention / retainer	Contenție / Retainer	Functional
38	Follow-up visit	Control	Functional
39	Crown (various types)	Coroană (tipuri diferite)	Functional
40	Corticotomy	Corticotomie	Functional
41	Laser periodontal therapy	Cura parodontala laser	Functional
42	Radical sinus surgery	Cura radicala sinus	Functional
43	Operculectomy	Decapușonare	Functional
44	Laser bacterial decontamination of periodontal pockets	Decontaminare bacteriana laser/ pungi parodontale	Functional
45	Dentatus screw post (brand)	Dentatus	Functional
46	Exposure / bonding of impacted tooth	Descoperire / ancorare dinte inclus	Functional
47	Desensitization / hypersensitivity treatment	Desensibilizare / Hipersensibilitate	Functional
48	Scaling	Detartraj	Functional
49	Periodontal pocket disinfection	Dezinfectie pungi parodontale	Functional
50	Root canal retreatment / removal of	Dezobturare / Îndepărtare instrument	Functional

	fractured instrument	din canal	
51	Microscope-assisted diagnosis	Diagnostic microscop	Functional
52	Rapid palatal expander	Disjunctoare	Functional
53	Distalizer / pendulum appliance	Distalizator / Pendulum	Functional
54	Endodontic drainage	Drenaj endodontic	Functional
55	Occlusal equilibration / TMJ therapy	Echilibrare ocluzală / ATM	Functional
56	Intermaxillary elastics	Elastice intermaxilare	Functional
57	Facial aesthetics (hyaluronic acid / botulinum toxin)	Estetică facială (acid hialuronic / botox)	Aesthetic
58	Periodontal assessment	Evaluare parodontala	Functional
59	Plaque disclosing	Evidentiere placa bacteriana	Functional
60	Histopathological examination	Examen anatomo patologic/ histopatologic	Functional
61	Periodic examination	Examinare periodica	Functional
62	Excision / biopsy of lesion	Excizie / Biopsie formațiune	Functional
63	Explantation	Explantare	Functional
64	Excision of tumoral lesions	Extirpare formatiuni tumorale	Functional
65	Tooth extraction	Extracție dentară	Functional
66	Dental veneer	Fațetă dentară	Aesthetic
67	Fibreglass post	Fibra sticla	Functional
68	Fluoridation	Fluorizare	Functional
69	Clinical photographs / photographic status	Fotografii / Status foto	Functional
70	Frenectomy / frenotomy	Frenectomie / Frenotomie	Functional
71	Germectomy	Germectomie	Functional
72	Surgical guide	Ghid chirurgical	Functional
73	Gingivectomy / gingivoplasty	Gingivectomie / Gingivoplastie	Functional
74	Gingivo-osteoplasty	Gingivo osteo plastie	Functional
75	Connective tissue graft	Grefa tesut conjunctiv	Functional
76	Occlusal splint / tray (various types)	Gutieră (diferite tipuri)	Functional
77	Headgear	Headgear	Functional
78	Haemostasis	Hemostaza	Functional
79	Professional oral hygiene	Igienizare profesională	Functional
80	Tooth splinting (trauma)	Imobilizare dinte (traumatism)	Functional
81	Dental implant	Implant dentar	Functional
82	Occlusal build-ups	Inaltari ocluzie	Functional
83	Perforation repair	Inchidere perforatie	Functional
84	Sinus closure	Inchidere sinus	Functional
85	Incision / abscess drainage	Incizie / Drenaj abces	Functional
86	Removal of orthodontic appliance	Indepartare aparat ortodontic	Functional
87	Foreign body removal	Indepartare corp străin	Functional
88	Removal of Dentatus screw post	Indepartare dentatus	Functional
89	Removal of intraradicular post / bone inlay	Indepartare ios	Functional
90	Inlay / onlay	Inlay / Onlay	Functional
91	Archwire replacement	Inlocuire arc	Functional
92	Band replacement	Inlocuire inel	Functional
93	Oral hygiene instruction	Instructiuni igiena bucala	Functional
94	Intraradicular post / bone inlay	Ios	Functional
95	Isolation (rubber dam)	Izolare (digă)	Functional
96	Alveolar nerve lateralization	Lateralizare nerv alveolar	Functional
97	Sinus lift	Lifting sinus	Functional
98	Definitive prosthetic restoration	Lucrare definitiva fast	Functional
99	Marsupialization	Marsupializare	Functional
100	Facemask (Delaire)	Mască facială (Delaire)	Functional
101	Periodontal measurements	Masuratori parodontale	Functional
102	Intracanal medication (calcium hydroxide)	Medicație intracanalară (hidroxid de calciu)	Functional
103	Membrane / healing screw	Membrană / Șurub vindecare	Functional
104	Space maintainer	Menținător de spațiu	Functional
105	Mock-up / wax-up (smile design)	Mock-up / Wax-up (design zâmbet)	Functional
106	Study models	Modele studiu	Functional
107	Mounting in articulator	Montare in articulator	Functional
108	Filling / restoration (caries)	Obturație / Plombă (carie)	Functional
109	Flap surgery	Operație cu lambou	Functional
110	Ozone therapy	Ozonoterapie	Functional
111	Laser treatment package	Pachet tratament laser	Functional

112	Periodontal charting / status	Parodontometrie / Status parodontal	Functional
113	Professional polishing	Periaj profesional	Functional
114	Palatal plate (protection / osteosynthesis)	Placuta palatinala protectie/ osteosinteza	Functional
115	Treatment plan	Plan de tratament	Functional
116	Closure of oro-antral communication	Plastie comunicare oro-sinusală	Functional
117	Pre-endodontic build-up	Pre endo build	Functional
118	Connective tissue harvesting	Prelevare tesut conjunctiv	Functional
119	Premolarization	Premolarizare	Functional
120	Platelet-rich fibrin (PRF)	PRF / Plasmă (fibrină)	Functional
121	Pulp vitality test	Proba vitalitate	Functional
122	Pulp protection	Protectie pulpara	Functional
123	Denture (various types)	Proteza (diferite tipuri)	Functional
124	Pulpectomy	Pulpectomie / Extirpare pulpară	Functional
125	Dental bridge	Punte dentară	Functional
126	Dental radiograph	Radiografie dentara	Functional
127	All-on-X implant-supported rehabilitation	Reabilitare All-on-X (pe implanturi)	Functional
128	Reattachment of tooth fragment	Reatasare fragment dentar	Functional
129	Band re-cementation	Recimentat inel	Functional
130	Re-bonding of buccal tube	Recolat tubusor	Functional
131	Post-and-core restoration	Reconstituire corono-radiculară (pivot/DCR)	Functional
132	Reconstruction	Reconstructie	Functional
133	Light-cured composite reconstruction	Reconstructie compozit fotopolimerizabil	Functional
134	Plaque reduction	Reducere placa bacteriana	Functional
135	Core build-up	Refacere bont coronar	Functional
136	Periodontal regeneration (enamel matrix derivative / GTR)	Regenerare parodontală (Emdogain/GTR)	Functional
137	Plaque disclosing	Relevare placă bacteriană	Functional
138	Denture repair / relining	Reparație / Rebazare proteză	Functional
139	Clasp repair	Reparație croșet	Functional
140	Tooth replantation	Replantare dentara	Functional
141	Alveolar nerve repositioning	Repozitionare nerv alveolar	Functional
142	Apicoectomy	Rezecție apicală	Functional
143	Digital scanning	Scanare digitala	Functional
144	Replacement of appliance components	Schimbare elemente aparat dentar	Functional
145	Appliance activation / repair visit	Ședință activare / reparație aparat	Functional
146	Fissure sealing	Sigilare șanțuri	Functional
147	Sinus lift	Sinus lift	Functional
148	Benefit distalization system (skeletal anchorage)	Sistem benefit distalizare	Functional
149	Irrigation / lavage	Spalaturi	Functional
150	Treatment planning	Stabilirea planului tratament	Functional
151	Interproximal stripping	Stripping	Functional
152	Suturing / suture removal	Sutură / Îndepărtare fire	Functional
153	Tooth jewellery (Skyce)	Tatuaj dentar / Skyce	Aesthetic
154	Telescopic crown	Telescop	Functional
155	Antimicrobial therapy	Terapie antimicrobiana	Functional
156	Laser therapy (decontamination / biostimulation / periodontal)	Terapie laser (decontaminare/biostimulare/ parodontala)	Functional
157	Microbiological testing	Testare microbiologica pet	Functional
158	Treatment of periodontal disease	Tratament bolii parodontale	Functional
159	Treatment of oral aphthae	Tratament afta bucala	Functional
160	Treatment of alveolitis (dry socket)	Tratament alveolită	Functional
161	Antibiotic treatment	Tratament antibiotic	Functional
162	Antiseptic treatment	Tratament antiseptic	Functional
163	Calcium hydroxide treatment	Tratament CaOH	Functional
164	Endodontic (root canal) treatment	Tratament endodontic (canal)	Functional
165	Treatment of pulp gangrene / necrosis	Tratament gangrenă / necroză	Functional
166	Treatment of gingivostomatitis	Tratament gingivostomatitei	Functional
167	Treatment of oral herpes	Tratament herpes	Functional
168	Treatment of dentine hypersensitivity	Tratament hiperestezie dentara	Functional
169	Laser treatment	Tratament laser	Functional
170	Treatment of incipient lesions (resin	Tratament leziuni incipiente	Functional

	infiltration / remineralization)	(ICON/remineralizare)	
171	Medication-based treatment	Tratament medicamentos	Functional
172	Treatment of molars / premolars	Tratament molari/premolari	Functional
173	Orthodontic treatment (other)	Tratament ortodontic (altul)	Functional
174	Periodontal treatment (curettage / root planing)	Tratament parodontal (chiuretaj/surfasaj)	Functional
175	Paediatric dental treatment	Tratament pedodonție	Functional
176	Treatment of pericoronitis	Tratament pericoronarită	Functional
177	Rotary root canal treatment	Tratament rotativ canal	Functional
178	Tuberoplasty	Tuberoplastie	Functional
179	Single-use turbine (consumable)	Turbina unica folosinta	Functional
180	Diagnostic wax-up	Up diagnostic	Functional
181	Emergency visit / dressing	Urgență / Pansament	Functional
182	Vestibuloplasty	Vestibuloplastie	Functional

Note: Entries correspond to the distinct service terms as advertised by the practices and include all listed items, including diagnostic and ancillary acts (e.g., consultation, radiography, treatment planning). Each item was classified according to the primary clinical purpose for which it is used — predominantly functional — rather than by procedure type, with ambiguous or mixed-indication items resolved by consensus. Because classification was applied to each listed term, closely synonymous wordings used by different practices (e.g., "Lifting sinus" and "Sinus lift") are retained as separate entries rather than merged, preserving fidelity to the source listings. Where a proprietary name was used in the source listing (e.g., Dentatus, Skyce, Benefit, Delaire), it is retained verbatim, with the generic descriptor given where applicable; no endorsement of any product or brand is implied.
